# Attention-Based Deep Recurrent Neural Network to Forecast the Temperature Behavior of an Electric Arc Furnace Side-Wall

**DOI:** 10.3390/s22041418

**Published:** 2022-02-12

**Authors:** Diego F. Godoy-Rojas, Jersson X. Leon-Medina, Bernardo Rueda, Whilmar Vargas, Juan Romero, Cesar Pedraza, Francesc Pozo, Diego A. Tibaduiza

**Affiliations:** 1 Departamento de Ingeniería Eléctrica y Electrónica, Universidad Nacional de Colombia, Cra 45 No. 26-85, Bogotá 111321, Colombia; dfgodoyr@unal.edu.co (D.F.G.-R.); dtibaduizab@unal.edu.co (D.A.T.); 2 Control, Modeling, Identification and Applications (CoDAlab), Department of Mathematics, Escola d’Enginyeria de Barcelona Est (EEBE), Campus Diagonal-Besòs (CDB), Universitat Politècnica de Catalunya (UPC), Eduard Maristany 16, 08019 Barcelona, Spain; jersson.xavier.leon@upc.edu; 3 Departamento de Ingeniería Mecánica y Mecatrónica, Universidad Nacional de Colombia, Cra 45 No. 26-85, Bogotá 111321, Colombia; 4 South32-Cerro Matoso S.A., Km 22 Highway SO Montelibano, Córdoba 234001, Colombia; Bernardo.S.Rueda@south32.net (B.R.); whilmar.p.vargas@south32.net (W.V.); JuanAlonso.Romero@south32.net (J.R.); 5 Departamento de Ingeniería de Sistemas e Industrial, Universidad Nacional de Colombia, Cra 45 No. 26-85, Bogotá 111321, Colombia; capedrazab@unal.edu.co; 6 Institute of Mathematics (IMTech), Universitat Politècnica de Catalunya (UPC), Pau Gargallo 14, 08028 Barcelona, Spain

**Keywords:** structural health monitoring, temperature forecasting, recurrent neural network, attention, GRU, LSTM, electric arc furnace

## Abstract

Structural health monitoring (SHM) in an electric arc furnace is performed in several ways. It depends on the kind of element or variable to monitor. For instance, the lining of these furnaces is made of refractory materials that can be worn out over time. Therefore, monitoring the temperatures on the walls and the cooling elements of the furnace is essential for correct structural monitoring. In this work, a multivariate time series temperature prediction was performed through a deep learning approach. To take advantage of data from the last 5 years while not neglecting the initial parts of the sequence in the oldest years, an attention mechanism was used to model time series forecasting using deep learning. The attention mechanism was built on the foundation of the encoder–decoder approach in neural networks. Thus, with the use of an attention mechanism, the long-term dependency of the temperature predictions in a furnace was improved. A warm-up period in the training process of the neural network was implemented. The results of the attention-based mechanism were compared with the use of recurrent neural network architectures to deal with time series data, such as Long Short-Term Memory (LSTM) and Gated Recurrent Unit (GRU). The results of the Average Root Mean Square Error (ARMSE) obtained with the attention-based mechanism were the lowest. Finally, a variable importance study was performed to identify the best variables to train the model.

## 1. Introduction

The control and monitoring of industrial processes require special attention because of their complexity, which is the result of the sub-processes and the multiple variables involved that need to be considered to know the current state of the general process. Regarding systems that make use of structures, the use of structural health monitoring (SHM) systems allows the proper monitoring of variables in the decision-making process, allowing better knowledge of the behavior of the structure and providing tools for maintaining tasks [1]. In an SHM system, some elements are required, such as the use of sensors permanently installed in the structure, a data acquisition system for sensing/actuating over the structure, a signal conditioning step, the development of statistical models and the possibility of a decision-making process [2]. This last element can be developed by computational tools in an autonomous way or by the analysis obtained from the statistical models. The literature includes multiple examples of developed monitoring systems and applications in different kinds of structures, such as those used in aircraft [3,4,5], buildings [6,7], bridges [8,9] and furnaces [10,11], among others.

Concerning furnace monitoring as used in smelting processes, the number of variables and the influence on the process is highly significant. As an example, in the case of the ferronickel production industry, this process can be performed in an electric arc furnace (EAF) [12] and the structural health monitoring (SHM) of the system requires the monitoring of several parts. The refractory hearth lining of an EAF is a crucial part to improve the campaign life of the furnace [13]. The lining monitoring variables comprise temperature, heat fluxes, water quality, remaining thickness refractory, sidewall erosion and protective layer formation, among others [14]. However, the development of temperature lining prediction models in an EAF is still an open research field because of the reduced number of works in this area [15,16].

Recently, the use of deep learning models has spread due to the data availability and their success rates in classification and regression tasks in minerals processing [17]. In addition, the success of deep learning models is based on their capacity for extracting features, improving the data-driven models in terms of accuracy and efficiency; moreover, the big data coming from a sensor network allow large-scale training based on deep learning models [18].

Cerro Matoso S.A. (CMSA) is one of the world’s major producers of ferronickel and it is operated by South32. This is an open-cut mine operation in Northern Colombia, /textcolorbluewith nearly 40 years of operation in the region. More details about the process developed by CMSA can be found directly on its web page https://www.cerromatoso.com.co/ (accessed on 10 January 2022 ). The complex process of produce ferronickel in the EAF of (CMSA) involves a number of variables. In this work, the lining temperature in an EAF is predicted using a multivariate time series deep learning model. The developed model is able to handle the multiple input variables as well as predict multiple thermocouple output variables. The time series approach was selected in order to process variable-length sequences of inputs. This kind of model can use recurrent neural networks (RNN) to handle the temporal dynamic behavior of the data. The long-term dependency of the temperature predictions in the EAF was compared using, first, a Long Short-Term Memory (LSTM) unit and, second, a Gated Recurrent Unit (GRU) approach [19]. These kinds of cells are used in contrast with traditional RNN due to the capacity to handle the vanishing and exploding long-term gradient problems [20]. The temporal information has been incorporated into deep learning models using different encoder architectures, such as convolutional neural networks (CNN), RNN and attention-based models [21]. Attention models allow us to identify relevant parts in the input sequence data to improve the prediction behavior of the deep learning model in the target time series [22,23,24].

The time series forecasting deep learning model is developed with data from a 75 MW shielded arc smelting furnace of CMSA [25]. This furnace is instrumented with a large set of thermocouples radially distributed in the lining furnace. The cooling system in the furnace uses plate and waffle coolers [26]. There are four levels of plate coolers radially distributed in 72 panels in the furnace.

The novelty of this work lies in the development of a time series forecasting deep learning model using an attention-based mechanism. This model takes into account as input variables different operation variables in the furnace, such as power, current, voltage, electrode position, amount of input material and chemistry composition. As output variables, 68 thermocouples radially distributed in the furnace lining were satisfactorily predicted at different forecast times in a range from 1 h to 6 h in the future.

The remainder of the paper is structured as follows. Section 2 includes the theoretical background, where all methods are described, followed by the dataset for validation in Section 3; then, the multivariate time series temperature forecasting model is described in Section 4. Then, the results and discussion are shown in Section 5, and, finally, the conclusions are included in the last section.

## 2. Theoretical Background

Here, the main concepts used in the development of the attention-based deep recurrent neural network model are described. For more information, the reader is referred to each provided reference.

### 2.1. Electric Arc Furnace

**Figure 1:**
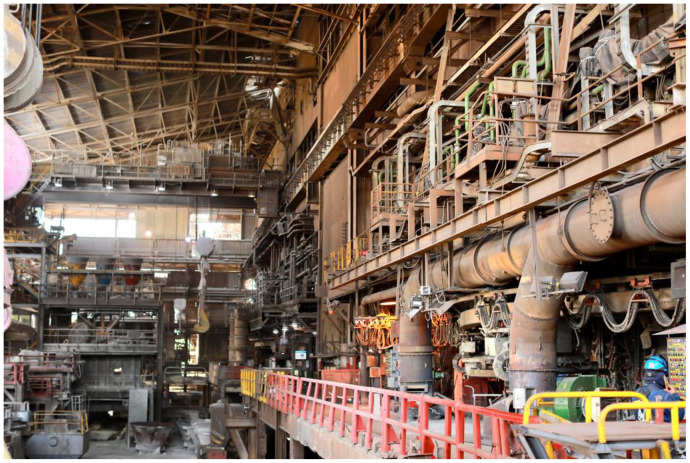
Panoramic of the CMSA plant.

The ferronickel production inside CMSA has several stages, including the mining and material homogenization phase, in which the material extracted from the mine is divided into smaller parts. Then, the phase of drying and storing the material is executed. Subsequently, the semi-dried material enters a rotatory kiln calciner; the material at the exit of this stage is called calcine, which is supplied to the electric arc furnace through different upper tubes distributed in three central, semi-central and lateral zones. The smelting stage is carried out within the EAF, which is detailed below. After the material is melted, it is ejected from the furnace employing two different runners, one for the ferronickel and the other for the slag. The next phase in the process is the refining and granulation phase of the material; finally, there is the finished product handling phase, where the material is packed and taken to commercialization. Figure 1 shows a picture of the building where the two furnaces are located. The dimensions of each furnace are 22 m in diameter and 7 m in height.

**Figure 2:**
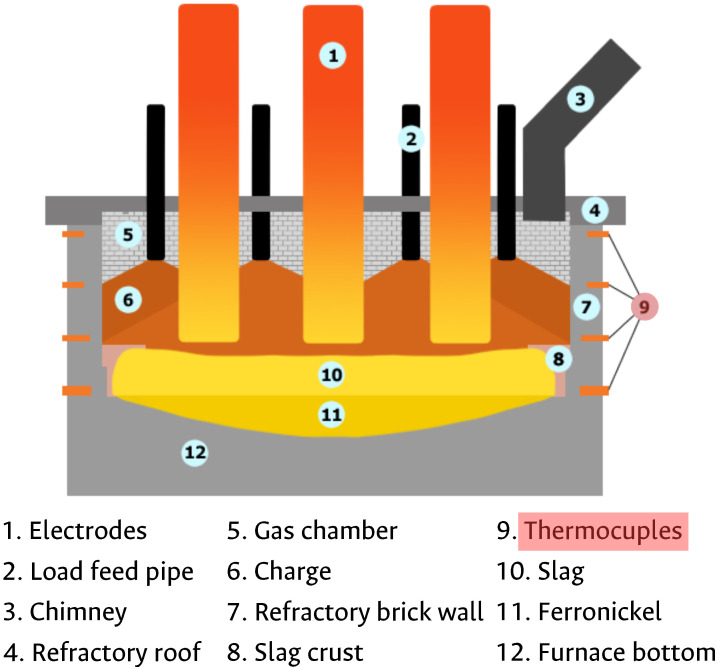
Electric arc furnace components description.

The main stage of ferronickel production is smelting. It is performed in the EAF. Figure 2 shows an inside view of the EAF, detailing its parts: (1) electrodes, (2) feeding tubes, (3) exhaust chimney, (4) top roof, (5) back-side wall, (6) input calcine, (7) sidewall, (8) waffle coolers, (9) plate coolers, (10) smelted ferronickel, (11) slag and (12) bottom hearth furnace lining. This study is focused on the temperature monitoring and forecasting of the side-wall; in particular, this temperature is measured by a thermocouples’ sensor network located at the plate coolers of the side-wall.

### 2.2. Multivariate Time Series Forecasting

The multivariate time series forecasting process seeks the behavior of a set of output variables at a specific future time. Several methods have been developed to model the relationships between fluctuating variables in time series data. These methods can be divided into classical and machine learning methods. Among the classical methods are Autoregressive Integrated Moving Average (ARIMA), Vector Autoregression (VAR) and Vector Autoregression Moving-Average (VARMA) [27]. In contrast to classical methods, machine learning methods are effective in more complex time series prediction problems with multiple input variables, complex nonlinear relationships and missing data [28]. Some machine learning algorithms used for regression tasks have been used for time series forecasting; among them are Support Vector Regression, Random Forest, Extreme Gradient Boosting and Artificial Neural Networks [21]. Recently, deep learning advances have emerged as a satisfactory method to perform time series forecasting. The recurrent neural networks and their variants, such as Gated Recurrent Unit (GRU) and Long Short-Term Memory (LSTM), have addressed the problem of vanishing gradient and long-term dependencies, achieving remarkable behaviors [19].

### 2.3. Encoder–Decoder

For each time step in the LSTM and GRU models, each input corresponds to one of the outputs. In some cases, the objective is to predict an output given a different-length input, without correspondence; the models developed for these cases are known as seq-to-seq models. A typical model has two parts, an encoder and a decoder, with two different networks combined into one network; this network can take an input sequence and generate the next most probable sequence as the output. First, the encoder traverses the input at each time step to encode the complete sequence in a vector called the context vector; this vector acts as the last hidden state of the encoder and as the first hidden state for the decoder. This will contain information about all the input elements, which will help in the realization of the predictions [29].

### 2.4. Attention Mechanism

One of the frontiers in deep learning is attention mechanisms, which represent an evolution of encoder–decoder models, which were developed to improve the performance of long input sequences. In attention mechanisms, the decoder can selectively access the encoded information and uses a new concept for the context vector c(t), which is now calculated at each time step of the decoder, from the previous hidden state and all the hidden states of the encoder [29]. Trainable weights will be assigned to these states and produce different degrees of importance to all the elements in the input sequence. Special attention is paid to the most significant inputs—hence, they are named attention mechanisms [30]. The construction of the context vector starts from the combination of each time step *j* of the encoder with each time step *t* of the decoder. This expression is called the alignment score, and it is calculated as follows:

(1) $$\mathrm{score}\left(j,t\right)=V_{a}\tan h\left(U_{a}s\left(t-1\right)+W_{a}h\left(j\right)\right)$$

The terms $V_{a}$, $W_{a}$ and $U_{a}$ correspond to the trainable weights mentioned above, where $V_{a}$ defines the function to calculate the alignment score, $W_{a}$ are associated with the hidden states of the encoder and $U_{a}$ with the hidden states of the decoder. The score must be normalized for each time step *t*; therefore, the SoftMax function is used together with the time steps *j*, and we obtain the attention weights α(j,t), defined as follows:

(2) $$\alpha \left(j,t\right)=\frac{e^{\mathrm{score}(j,t)}}{\sum_{j=1}^{M}e^{\mathrm{score}(j,t)}}$$

This weight can capture the importance of the input at time step *j* to adequately decode the output at time step *t*. Finally, the context vector is found from the weighted sum of the relationship between all the encoder hidden values and attention weights:

(3) $$c\left(t\right)=\sum_{j=1}T\alpha \left(j,t\right)h\left(j\right)$$

The context vector allows more attention to the relevant inputs in the electric arc furnace variables. The term c(t) passed through the decoder and the probability for the next possible output is calculated. This operation applies to all time steps at the input. Then, the current hidden state s(t) is calculated, taking as input the context vector c(t), the previous hidden state s(t−1) and the output $\hat{y}\left(t-1\right)$ from the previous time step:

(4) $$s\left(t\right)=f(s\left(t-1\right),\hat{y}\left(t-1\right),c\left(t\right))$$

Therefore, using this attention mechanism, the model can find the correlations between the different parts of the input sequence to the corresponding parts of the output sequence. For each time step, the decoder output is calculated by applying the “SoftMax” function to the hidden state [31].

### 2.5. Root Mean Squared Error (RMSE)

The performance of the multivariate time series forecasting deep learning model is calculated using the Root Mean Squared Error (*RMSE*):

(5) $$RMSE=\sqrt{\frac{1}{B}\sum_{i=1}^{B}{\left(\hat{y_{i}}-y_{i}\right)}^{2}}$$

where *B* is the number of data points in the time series to be estimated, $y_{i}$ is the actual value of the time series, and $y_{i}$ is the estimated value at the time *i* by the prediction model.

## 3. Dataset for Validation

**Figure 3:**
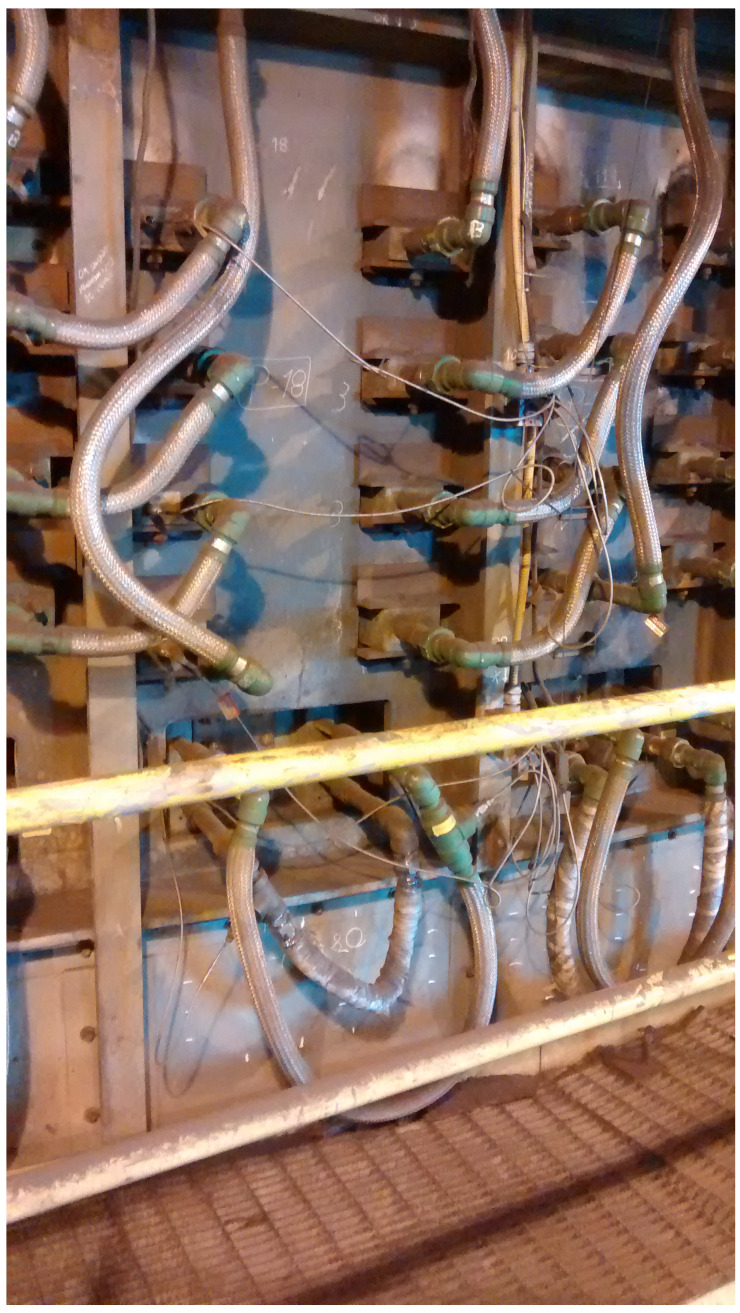
Photography of CMSA furnace outside wall with its coolers.

Data used to train and validate the attention-based deep RNN model were obtained from a thermocouple sensor network located at the side-wall of an EAF in CMSA. Photography of the EAF side-wall is shown in Figure 3. The EAF side-wall is composed of 72 radially distributed panels. Figure 3 details a portion of the side-wall of 1 panel. The illustrated hoses carry water, which is used to cool the refractory walls of the EAF through the plate coolers (4 for each panel) and the waffle cooler (1 for each panel).

**Figure 4:**
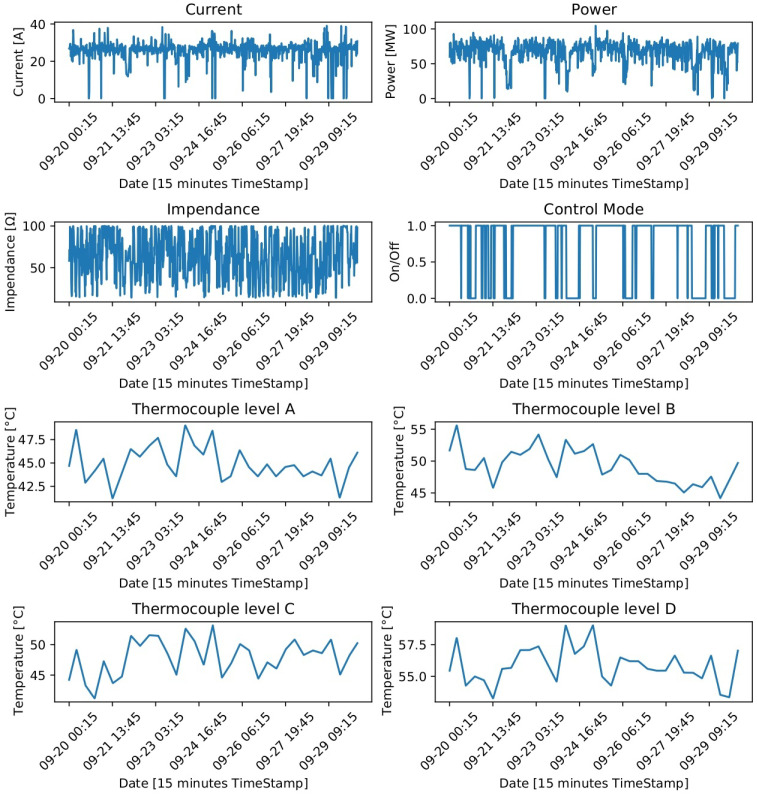
Time series plot of some input and output variables in the dataset.

The dataset used for model training and validation is composed of data recorded during 5 years, with 177,312 instances and 49 attributes, from an EAF located in Cerro Matoso, South 32 company. Data were collected every 15 min during a period of 1847 days, from September 9th of 2016 to September 30th of 2021. The input variables in the model were related to electrode current, voltage, arc, power, calcine feed, the chemical composition of the calcine, relative electrode position and 16 thermocouples. These 16 thermocouples were also taken as output variables to predict. In particular, 4 panels radially distributed 90 degrees in each quadrant of the furnace were selected to study the behavior of their plate cooler thermocouples. Each of the selected 4 panels had 4 plate coolers; thus, a total of 16 plate coolers were analyzed. The behavior of the time series of some of these variables in a time window that allows the trend to be seen can be observed in Figure 4.

Several data preprocessing steps were performed to detect abnormal behavior in the used variables. These data preprocessing steps are listed below [32]:

- Remove duplicates;
- Treat empty and null values;
- Treat unique values;
- Encode strings;
- Remove negative temperatures;
- Eliminate variables with high variance;
- Remove variables with zero variance.

After verifying the data preprocessing, it was concluded that the 49 variables used to train and test the models did not present abnormal behaviors.

## 4. Multivariate Time Series Temperature Forecasting Model

**Figure 5:**
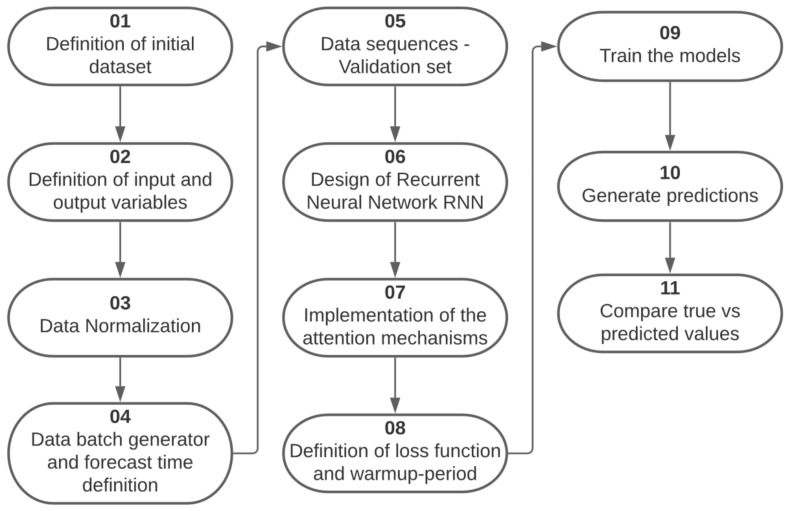
Step by step development of the temperature prediction model.

The development of the multivariate time series temperature forecasting model comprised several stages. It started with the definition of the initial set of data already preprocessed, where the input variables for the models were selected as well as the variables to be predicted, the data were normalized so that the neural networks could work with them, the forecast time was defined and, in this way, the batch set generator was created for model training, as well as the data sequences for validation. The neural network models GRU and LSTM are designed to be incorporated with the attention mechanisms, and the RMSE loss function is defined with a warm-up period of 50 steps, which was not considered for the calculation of the evaluation metric, so we proceeded to train the model, validate it and generate the predictions to be able to compare them with the real values and make conclusions; this process is summarized in Figure 5.

**Figure 6:**
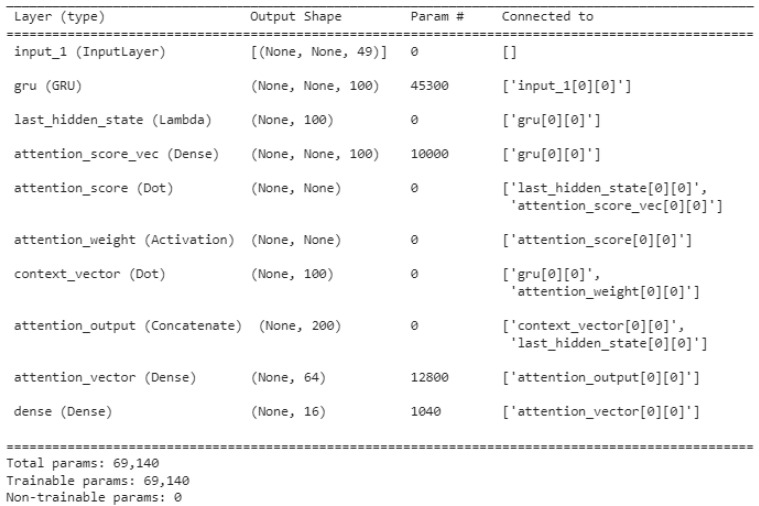
Details of the shape and layer connections in the attention-based multivariate time series forecasting model.

The different layers that compose the multivariate time series deep learning attention models are summarized in Figure 6. Details of the shape and the number of parameters and connections of the layers in the multivariate time series deep learning attention model are noted. For the the gradient descent method, we used Adam optimization; this is a stochastic gradient descent (SGD) method that is based on adaptive estimation.

## 5. Results and Discussion

Four deep neural network configurations corresponding to a GRU model and an LSTM model, with and without attention mechanisms, were designed, trained and tested, using 49 input variables to predict the 16 output variables corresponding to the thermocouple temperature. The Average Root Mean Square Error (RMSE) of these 16 output variables was used as a performance metric for each of the models.

### 5.1. Influence of Changing the Prediction Time

**Figure 7:**
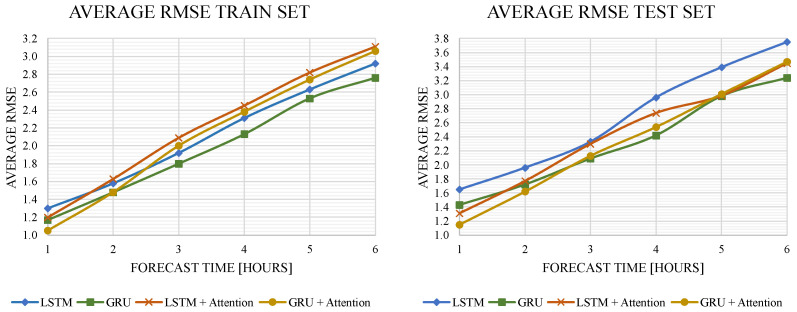
Average RMSE behavior over the forecast time increases.

To determine the models’ behavior relating to the time interval under which they performed the prediction, the test was performed in a time window of 1 to 6 h predicting in the future for each model, increased by 1 h, as shown in Table 1 and Figure 7. From Table 1, it is evident that the Average RMSE values in the test set are larger than the train values. This is caused by the large amount of data belonging to the train set (90%) compared to the data from the test set (10%).

Figure 7 shows that the models with attention mechanisms had higher performance during a shorter prediction time. As the prediction time increased from 1 to 6 h, the models without attention mechanisms outperformed the other models. The GRU model obtained the best results with attention mechanisms for short times and without attention mechanisms for long times; for the short times, the longer input sequence in the GRU and LSTM networks resulted in worse prediction accuracy of the output sequence because it focused on all input variables equally. An attention mechanism can be used to alleviate this problem by focusing on more relevant input variables, since, as already described above, attention mechanisms can adaptively assign a different weight to each input sequence to automatically choose the most relevant features of the time series. Therefore, the model can effectively capture the long-term dependence on the time series.

**Figure 8:**
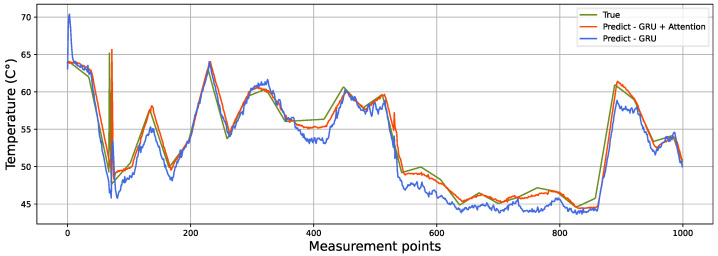
Predictive versus true behavior of the GRU and GRU + attention models in the test set in one of the output thermocouples.

As a result of the models evaluated in a 1 h forecast with and without attention, the predicted and true behaviors for a single thermocouple were compared, as shown in Figure 8. It is evident that the GRU model including attention (orange line) obtained a better representation of the true (green line) behavior. In contrast, the only GRU model (blue line) presented a more curly and distant behavior from the true data.

Additionally, in Table 2, the individual comparison of the RMSE error of each one of the thermocouples for each model used is presented. Here, it can be observed how some thermocouples have small prediction errors and others very large, which is averaged and leads to obtaining the Average RMSE of the total forecast.

### 5.2. Parameter Exploration

To evaluate the influence of some parameters in the Average RMSE results, an exploration procedure was executed. The changing of three different parameters was evaluated. These parameters were the optimizer, the number of cells in the GRU and LSTM models and finally the number of training epochs in the GRU model.

#### 5.2.1. Changing of Optimizer

Four different optimizers were evaluated in order to compare their influence on the Average RMSE obtained by the GRU model. The four compared optimizers were RMSprop, Adam, Adamax and Nadam. As shown in Table 3, the best optimizer was Adam, obtaining an RMSE value in the train set of 3.01.

#### 5.2.2. Change of GRU Cell Number

The variation in the number of GRU cells was studied by changing this number from 50 to 175, as shown in Table 4. The results indicate that, as the number of cells increases, the RMSE in the training set decreases, which does not mean that it is a good result because, in this way, the model is over-fitting with the training data, which means that, as the number of cells increases, the RMSE of the test set becomes worse because the model is so adjusted to the training data that when new and unknown data arrive in the model input, it is more difficult to make an adequate prediction.

#### 5.2.3. Change of LSTM Cell Number

Three different cell numbers were compared in the LSTM model. In this case, they were 32, 64 and 96, as shown in Table 5. The results show favorable behavior for the variation of 64 cells; as in the GRU model, more units does not lead to better results, due again to phenomena such as over-fitting.

#### 5.2.4. Changing of the Loss Behavior of the GRU Model through the Epochs

**Figure 9:**
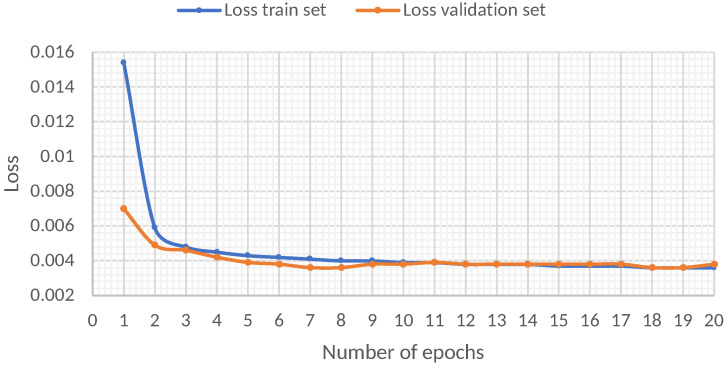
Evaluation of the loss behavior of the GRU model through the epochs in the train set and test set.

Figure 9 shows the loss behavior as the number of training epochs increases. From the results, it is evident that the first seven epochs are crucial in the decrease in loss, while, from epoch 7 onwards, the decrease in loss is scarce.

### 5.3. Time Series Cross-Validation

Different cross-validation procedures have been developed to evaluate the behavior of a time series forecasting model [33]. In this study, three different approaches to perform time series cross-validation were used. These three approaches were (a) 7-fold moving origin, (b) Blocking Time Series Split and (c) Blocking Time Series Split with a static test set. Below, these three approaches are described and discussed.

#### 5.3.1. Seven-Fold Moving Origin Time Series Split Cross-Validation

**Figure 10:**
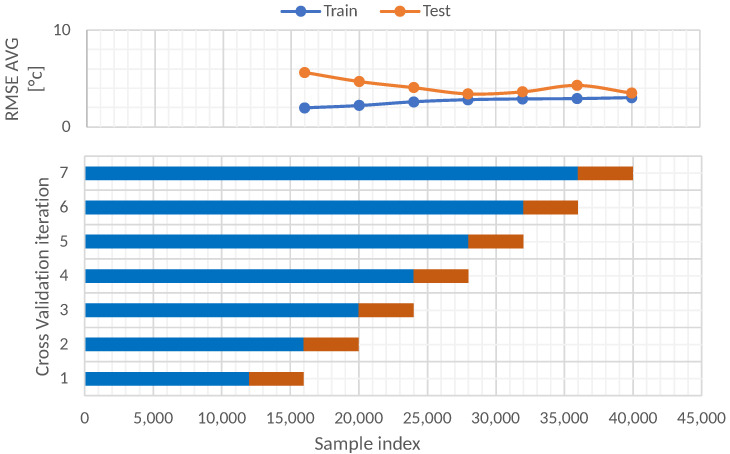
Time series split cross-validation data partitions.

The first approach for the time series cross-validation was the 7-fold moving origin. This procedure involves cumulative training data from 1 October of 2020 to 1 September of 2021. Figure 10 illustrates the results at the top and details the data division in the bottom section. Seven different folds were evaluated; the first is the least in the training set, and as the folds increase, the size of the training data also increases. The size of the test set remains constant in each fold. The size of this test set is 4000 data instances.

Table 6 shows the RMSE results for the train and test sets in each fold. As can be seen, the train RMSE increases as the number of folds increases. The opposite happens with the behavior of the RMSE test set; this indicates that it is better to train with numerous data because, with more data, the model can learn different scenarios that are presented in the furnace.

#### 5.3.2. Blocking Time Series Split

**Figure 11:**
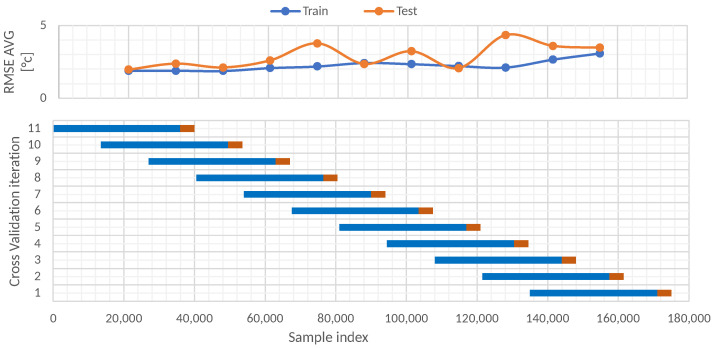
Blocking time series split cross-validation data partitions.

A second study using a blocking time series split cross-validation was performed. This validation approach consists of setting a fixed size of the train and test sets and moving across the entire dataset in several folds. In this case, 11 folds were used, and the train test had a size of 36,000 instances, whereas the test size had a size of 4000 instances. The shift between each fold was 140 days. Figure 11 illustrates the 11 folds and every train set in blue and test set in orange. In total, 177,312 instances of the dataset were used; these data began on 9th September of 2016 and ended on 30th September of 2021.

The results after performing the 11-fold blocking time series split cross-validation are shown in Table 7. From these results, it is evident that the best results of RMSE in the train set (1.89) and test set (1.96) were reached by the oldest fold—in this case, the 11th fold. Furthermore, a decreasing behavior of the RMSE through the folds is evident for the train set. In contrast, the behavior of the test set is oscillatory decreasing. Considering the 11 folds, the average RMSE was 2.24 for the train set and 2.89 for the test set.

#### 5.3.3. Blocking Time Series Split with Static Test Set

**Figure 12:**
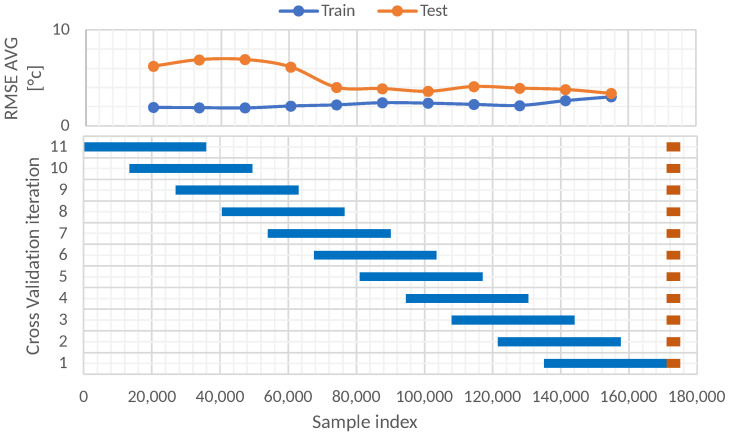
Blocking time series split with static test set cross-validation data partitions.

The last study for the time series cross-validation model was the blocking time series split with the static test set. In this case, 11 folds were also evaluated, but the test set remained the same for every fold. This test set was created with the most recent 4000 instances. Different training sets were tested. The shift between each training fold was 140 days. The size of each training set was 36,000 instances. Figure 12 illustrates the blocking time series split with static test set approach.

The RMSE results of the blocking time series split with static test set approach are shown in Table 8. The results indicate that it is preferable to perform training with recent data since the RMSE increases as the training data move away from the test data. The RMSE in the test set changes from 3.37 for the first fold to 6.21 in the 11th fold, which represents an increase of 84.27%. Therefore, it is advisable to train the model every certain period to avoid obvious increases in the RMSE.

### 5.4. Variable Importance Study

**Figure 13:**
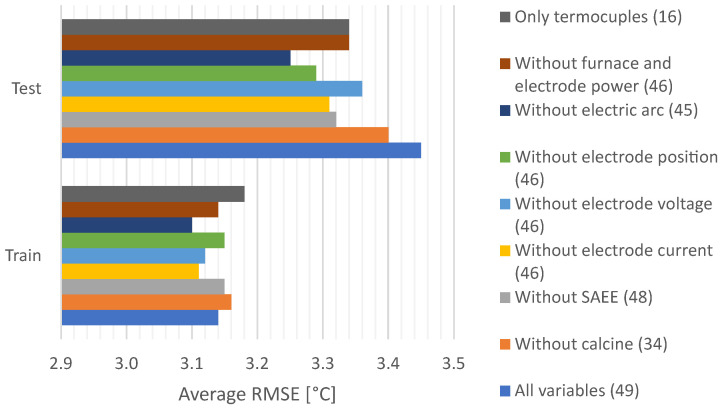
Variable influence in the Average RMSE of the GRU model.

A study of the selected variables used to train and test the GRU model was performed to evaluate their influence on the RMSE values. This study was performed with the data of the 7th fold in the time series split cross-validation data partitions shown in Figure 10. Therefore, 36,000 instances composed the training set, whereas 4000 instances constituted the test set. Seven different scenarios were selected to train and test, removing the original number of variables as follows: (a) only with the 16 thermocouples to predict, (b) without furnace and electrode electric power, (c) without an electric arc, (d) without electrode position, (e) without electrode voltage, (f) without electrode current, (g) without the automatic control of electric power in the furnace (SAEE) mode, (h) without calcine chemistry and (i) using all 49 variables. The results of the RMSE comparison are shown in the bar chart of Figure 13. From the results in the bar chart, one can observe the difference between the RMSE results in the train and test sets, the latter being the one with the largest RMSE values. In particular, the worst results in the test set were obtained by the (i) all variables’ configuration, causing the error to reach the highest value of 3.45 in the test set. Consequently, when removing different groups of variables, the RMSE value improved. The lowest RMSE value of the test set of 3.22 was reached when the group (c) without electric arc was removed. Thus, it is better to remove the group of variables (c) related to the electric arc to develop the GRU model.

### 5.5. Study of Increasing the Number of Predicted Thermocouples to 76

Based on a request made by the CMSA engineering team in which they preferred to concentrate on the monitoring of the plate coolers in the lower row, the number of thermocouples to be monitored and predicted was increased. A study of the increase in the number of thermocouples was carried out, progressively evaluating their impact on the Average RMSE of the predictions.

**Figure 14:**
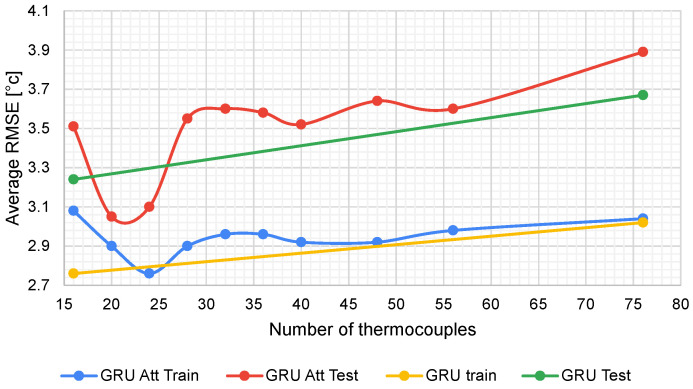
Average RMSE behavior when increasing the output thermocouples to predict.

The number of thermocouples was progressively increased from 16 until reaching 76 thermocouples. The RMSE values of the training set and the test set were measured for each increase; these values are shown in Table 9. It can be seen that the relationship between the increase in thermocouples and the Average RMSE of the predictions is directly proportional since, in Figure 14, the increasing trend of this evaluation metric can be observed due to the increase in the number of variables to be predicted.

The increase that occurs in the Average RMSE is small compared with the increase in the number of thermocouples. There was an increase of 4.75 times in the number of thermocouples, while the RMSE in the training set remained approximately constant because there was more information that the model could use to obtain better relationships between the variables. On the other hand, for the test set, the RMSE increased only 1.1 times; this is because the number of variables and data that the model must predict is greater, but it is still a good prediction result.

From the results depicted in Figure 14, it can be seen that the attention GRU model improved the RMSE value when 24 thermocouples were predicted. Therefore, for a few thermocouples, the attention GRU model is better, whereas, for numerous thermocouples, it is recommended to use the GRU model without the attention mechanism.

### 5.6. Root Mean Squared Error Distribution by Each Thermocouple in the Test Set

**Figure 15:**
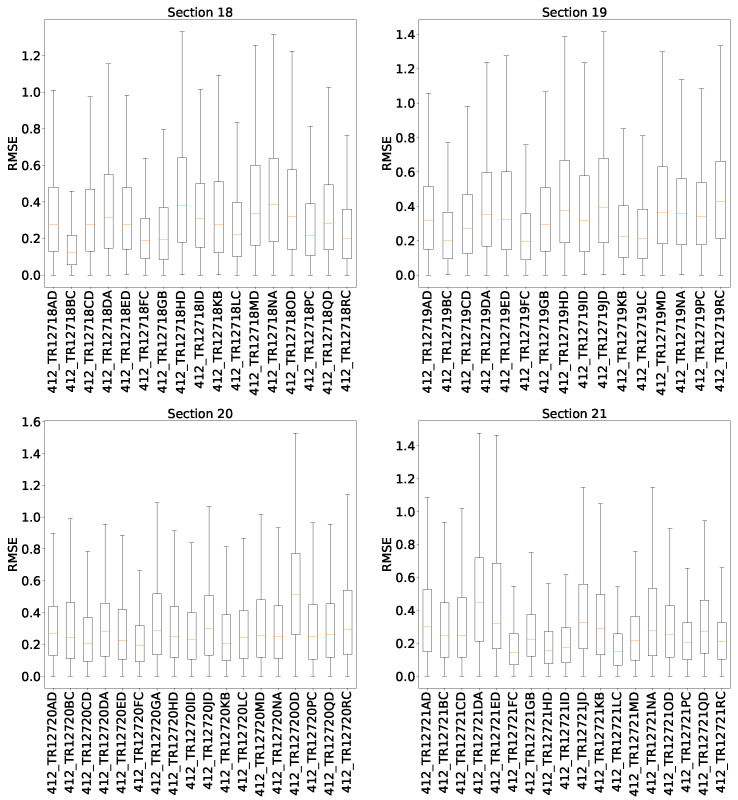
Absolute error analysis by each thermocouple in the test set.

Figure 15 illustrates the boxplot of the RMSE obtained by each thermocouple in the test set. In particular, the four quadrants of the furnace are separated. These quadrants are named as follows: northwest (section 18), southwest (section 19), southeast (section 20) and northeast (section 21). From the results, the southeast quadrant presents the lower error, while the southwest quadrant presents the highest. In general, the mean value of RMSE for each thermocouple is near to 0.4, reaching a maximum value of 1.75.

## 6. Conclusions

This work has shown the development of a multivariate time series deep learning model to predict the temperature behavior of a lining furnace. The developed model is based on an attention mechanism in the encoder–decoder approach of a recurrent neural network. The validation of the model was performed using data acquired in an industrial ferronickel furnace over a period of 5 years. The model considered the historical behavior of 49 variables involved in ferronickel production. Among these variables were the electrode current, voltage, power and position, besides the electric arc, the chemical composition and the temperature measured by the thermocouples themselves. These results were validated by a study carried out in terms of the Average RMSE calculated in 76 different thermocouples located in the furnace lining side-wall at four different heights.

The principal conclusions of this work are as follows:

- The temperature of the lining furnace at different heights of the wall and in different sectors was satisfactorily predicted using the developed deep learning model.
- The results showed that the prediction time influenced the obtained Average RMSE, which was better when predicted in a time window of 1 h in the future when the attention mechanism was used. RMSE values increased as the time window increased.
- A comparison between four different approaches using GRU, LSTM, and their attention-based variants was performed. The best RMSE results were obtained using the GRU attention-based model.
- Three different time series cross-validation procedures were used: the 7-fold moving origin time series split, the Blocking Time Series Split and the Blocking Time Series Split with static test set. The results showed that, over time, the model lost its ability to correctly predict temperatures. Therefore, it is recommended to retrain the model every year to maintain an RMSE value of around 4 °C.
- A study increasing the number of thermocouples to predict from 16 to 76 was carried out. The results showed that the Average RMSE was maintained at a value near to 4 °C, which is allowed in the furnace operation due to the normal operation conditions.

As general conclusions, we can highlight that this work aimed to provide and validate a forecast temperature methodology that is applied to an electric arc furnace. The validation was performed by using real data from a furnace of the Cerro Matoso S.A. and results were validated by staff from the same company. Although the methodology was implemented in this furnace, the paper presents the steps to apply it to any multivariable process to predict the behavior of a variable.

As future works, the following ideas will be explored:

- An online learning-based stream data approach will be developed to evaluate damages in the refractory walls using the developed model. Moreover, the concept of drift detection and treatment in these variables will be studied.
- The methodology will be adapted to forecast other important variables in this furnace, such as the thickness of the refractory wall by predicting, among others, the flow heat in these walls. Since thickness can be measured directly by the operational conditions, it can be obtained by a model that uses forecasted variables.

## Figures and Tables

**Table 1:** Average RMSE results of the train and test sets for the four different deep learning models in 6 different times.

MODEL	SET	AVERAGE RMSE TRAIN—TEST SETS [°C]					
		1 H	2 H	3 H	4 H	5 H	6 H
LSTM	Train	1.30	1.58	1.92	2.31	2.63	2.92
	Test	1.65	1.96	2.33	2.96	3.39	3.75
GRU	Train	1.17	1.48	1.80	2.13	2.53	2.76
	Test	1.43	1.72	2.09	2.42	2.98	3.24
LSTM + Attention	Train	1.20	1.63	2.09	2.45	2.82	3.11
	Test	1.31	1.77	2.30	2.74	2.99	3.45
GRU + Attention	Train	1.05	1.48	2.00	2.38	2.74	3.06
	Test	1.15	1.62	2.13	2.54	3.01	3.47

**Table 2:** RMSE results in the 16 thermocouples for the train and test sets versus the different deep learning models for a 6 h forecast.

	GRU		GRU + ATT		LSTM		LSTM + ATT	
**Thermocouple (T)**	**Train**	**Test**	**Train**	**Test**	**Train**	**Test**	**Train**	**Test**
T1	2.11	3.62	2.07	3.38	1.69	3.77	2.13	3.65
T2	2.68	3.12	2.76	3.18	2.18	3.51	2.91	3.15
T3	2.05	2.40	2.31	2.37	1.56	2.61	2.32	2.56
T4	1.39	1.12	1.46	1.15	1.22	1.29	1.47	1.15
T5	3.22	3.87	3.59	4.13	2.88	4.35	3.59	4.16
T6	3.69	4.60	3.76	4.23	3.08	5.02	3.80	4.22
T7	2.33	3.46	2.65	2.94	1.83	3.82	2.66	3.03
T8	1.58	1.23	1.62	1.25	1.38	1.48	1.66	1.24
T9	2.21	2.38	2.43	2.44	1.98	2.44	2.46	2.48
T10	2.49	2.26	2.65	2.46	2.10	2.52	2.65	2.74
T11	2.29	2.70	2.57	2.89	1.81	3.42	2.62	2.78
T12	1.56	1.40	1.65	1.33	1.38	1.62	1.69	1.41
T13	7.31	8.22	7.26	8.03	6.54	5.83	7.44	8.05
T14	6.63	6.65	6.71	6.50	6.35	7.32	6.78	6.55
T15	4.16	5.14	4.25	4.43	3.72	5.66	4.29	4.47
T16	2.86	3.29	2.72	2.63	2.57	2.99	2.83	3.11

**Table 3:** Average RMSE for GRU model with attention mechanisms against optimizer variance.

SET	Optimizer			
	RMSprop	Adam	Adamax	Nadam
Train RMSE	3.08	3.01	3.13	3.03
Test RMSE	3.62	3.32	3.37	3.44

**Table 4:** Average RMSE for GRU model with attention mechanisms against GRU unit variance.

SET	GRU UNITS					
	50	75	100	125	150	175
Train RMSE	3.11	3.08	3.06	3.05	3.04	3.03
Test RMSE	3.41	3.26	3.29	3.31	3.35	3.39

**Table 5:** Average RMSE for LSTM model with attention mechanisms against LSTM unit variance.

SET	LSTM UNITS		
	32	64	96
Train RMSE	3.25	3.10	2.80
Test RMSE	3.44	3.41	3.81

**Table 6:** Average RMSE for training and test sets at each iteration with time series split.

# Iteration	Train RMSE	Test RMSE
1	1.99	5.63
2	2.22	4.70
3	2.60	4.07
4	2.81	3.43
5	2.89	3.64
6	2.92	4.30
7	3.02	3.51

**Table 7:** Average RMSE for training and test sets at each iteration with blocking time series split.

# Iteration	Train RMSE	Test RMSE
1	3.08	3.46
2	2.65	3.59
3	2.11	4.33
4	2.21	2.06
5	2.34	3.22
6	2.42	2.35
7	2.18	3.76
8	2.07	2.59
9	1.87	2.11
10	1.89	2.37
11	1.89	1.96
Average RMSE	2.24	2.89

**Table 8:** Average RMSE for training and test sets at each iteration, with blocking time series split with static test set.

# Iteration	Train RMSE	Test RMSEr
1	3.02	3.37
2	2.62	3.78
3	2.13	3.92
4	2.24	4.09
5	2.36	3.59
6	2.41	3.88
7	2.19	4.01
8	2.07	6.13
9	1.87	6.91
10	1.90	6.89
11	1.92	6.21
Average RMSE	2.24	4.79

**Table 9:** Average RMSE results when increasing the number of thermocouples to predict in the GRU attention model.

Number of Thermocouples	GRU Att Train	GRU Att Test
16	3.08	3.51
20	2.90	3.05
24	2.76	3.10
28	2.90	3.55
32	2.96	3.60
36	2.96	3.58
40	2.92	3.52
48	2.92	3.64
56	2.98	3.60
76	3.04	3.89

## Data Availability

Not applicable.

## Abbreviations

The following abbreviations are used in this manuscript:

RMSE	Root mean square error
RNN	Recurrent neural network
LSTM	Long short-term memory
GRU	Gated recurrent unit
SHM	Structural health monitoring
EAF	Electric arc furnace
CNN	Convolutional neural network
CMSA	Cerro Matoso S.A.
